# Do hotspots fuel malaria transmission: a village-scale spatio-temporal analysis of a 2-year cohort study in The Gambia

**DOI:** 10.1186/s12916-018-1141-4

**Published:** 2018-09-14

**Authors:** Gillian H. Stresman, Julia Mwesigwa, Jane Achan, Emanuele Giorgi, Archibald Worwui, Musa Jawara, Gian Luca Di Tanna, Teun Bousema, Jean-Pierre Van Geertruyden, Chris Drakeley, Umberto D’Alessandro

**Affiliations:** 1 Department of Immunology and Infection, London School of Hygiene and Tropical Medicine, London, UK; 2 Medical Research Council Unit The Gambia at London School of Hygiene & Tropical Medicine, Fajara, The Gambia; 3 University of Antwerp, Antwerp, Belgium; 4 CHICAS, Lancaster Medical School, Lancaster University, Lancaster, UK; 5 Queen Mary University of London, London, UK; 6 Department of Medical Microbology, Radboud Medical University, Nijmegen, The Netherlands

**Keywords:** Hotspot, Foci, Geostatistics, Cohort, Spatial epidemiology

## Abstract

**Background:**

Despite the biological plausibility of hotspots fueling malaria transmission, the evidence to support this concept has been mixed. If transmission spreads from high burden to low burden households in a consistent manner, then this could have important implications for control and elimination program development.

**Methods:**

Data from a longitudinal cohort in The Gambia was analyzed. All consenting individuals residing in 12 villages across the country were sampled monthly from June (dry season) to December 2013 (wet season), in April 2014 (mid dry season), and monthly from June to December 2014. A study nurse stationed within each village recorded passively detected malaria episodes between visits. *Plasmodium falciparum* infections were determined by polymerase chain reaction and analyzed using a geostatistical model.

**Results:**

Household-level observed monthly incidence ranged from 0 to 0.50 infection per person (interquartile range = 0.02–0.10) across the sampling months, and high burden households exist across all study villages. There was limited evidence of a spatio-temporal pattern at the monthly timescale irrespective of transmission intensity. Within-household transmission was the most plausible hypothesis examined to explain the observed heterogeneity in infections.

**Conclusions:**

Within-village malaria transmission patterns are concentrated in a small proportion of high burden households, but patterns are stochastic regardless of endemicity. Our findings support the notion of transmission occurring at the household and village scales but not the use of a targeted approach to interrupt spreading of infections from high to low burden areas within villages in this setting.

**Electronic supplementary material:**

The online version of this article (10.1186/s12916-018-1141-4) contains supplementary material, which is available to authorized users.

## Background

Within populations, heterogeneity in exposure to malaria has been widely documented; it is generally estimated that 20% of the population experience 80% of the disease burden [1–3]. The skewed distribution of exposure has been observed at every spatial scale, in different transmission landscapes, and is expected to be more pronounced when transmission is low [4]. Several studies have documented both spatial and spatio-temporal high burden areas of malaria, typically referred to as hotspots but here defined as clusters, and have fueled the notion of spatially targeting interventions for control and elimination [5–7].

The consistent presence of spatial clusters of high malaria burden within populations contributed to the hypothesis that there may be hotspots, or certain households, or subsets of households within foci (spatially discrete areas with sustained transmission) that fuel transmission [8]. The number and size of clusters within foci and the delineation of a foci itself will likely depend on the specific setting. For example, on the coast of Kenya, multiple clusters were identified per foci [2], whereas a single cluster was observed in a highland setting [6]. If such clusters are in fact hotspots, meaning they are drivers of malaria transmission, and they could be easily identified and targeted with interventions, then resources could be used more effectively and their impact on transmission intensity may be greater than that of a uniform approach [8, 9]. For a hotspot-driven approach at the sub-village level to be viable, it is critical to determine whether the observed heterogeneity at the village scale is a feature of malaria transmission and supports the notion of “hotspots” fueling transmission or whether it follows a more stochastic pattern [10].

The notion of hotspots as intrinsic drivers of malaria transmission being an inherent part of the transmission landscape is plausible with risk being driven by macroscale and microscale characteristics [11, 12]. For example, the observed seasonality in transmission is associated with climate, specifically the rainfall patterns and temperature [13, 14]. Similarly, at the local scale, malaria risk is known to be associated with microepidemiological variation in risk factors, including greater odds of infection in those residing in proximity to mosquito breeding sites (e.g., ponds or forests) or living with other infected individuals [15–17]. The observed spatial heterogeneity in infected individuals also has implications for quantifying and understanding transmission intensity [18]. As described as part of the hotspot model, the high burden households within an endemic area may amplify transmission by acting as a constant parasite reservoir, or equally they could absorb infectious bites, attenuating observed transmission events [19, 20]. If these households or groups of households are driving transmission within foci, then hotspot-targeted interventions would be justified [8, 21].

Although biologically plausible, the evidence to support the concept of hotspots, here considered as a single household or group of high burden households within foci, fueling transmission has been mixed. For example, a recent trial targeting serologically defined hotspots of exposure failed to observe any sustained reduction in transmission outside of the targeted area [22]. Transmission in the study area may have been too high for well-defined hotspots, hotspot boundaries may not have been effectively defined, or hotspots may not have contributed to maintaining transmission in this setting [23]. Despite the limited evidence to support the use of hotspot-targeted approaches, several malaria elimination programs have engaged in hotspot-inspired strategies [3, 21, 24].

In this study, we conducted a spatio-temporal analysis on a full population cohort distributed in six pairs of villages across The Gambia. The aim of this research was to establish if predicted risk of malaria transmission intensity exhibits a consistent pattern, meaning the risk of malaria moving from a high burden household or a group of households to neighboring households, over time. If the expected pattern exists, we aimed to identify at what transmission intensity this dynamic becomes apparent. In case of limited evidence to support the hotspot pattern, some potential drivers of any observed heterogeneity were explored.

## Methods

**Fig. 1:**
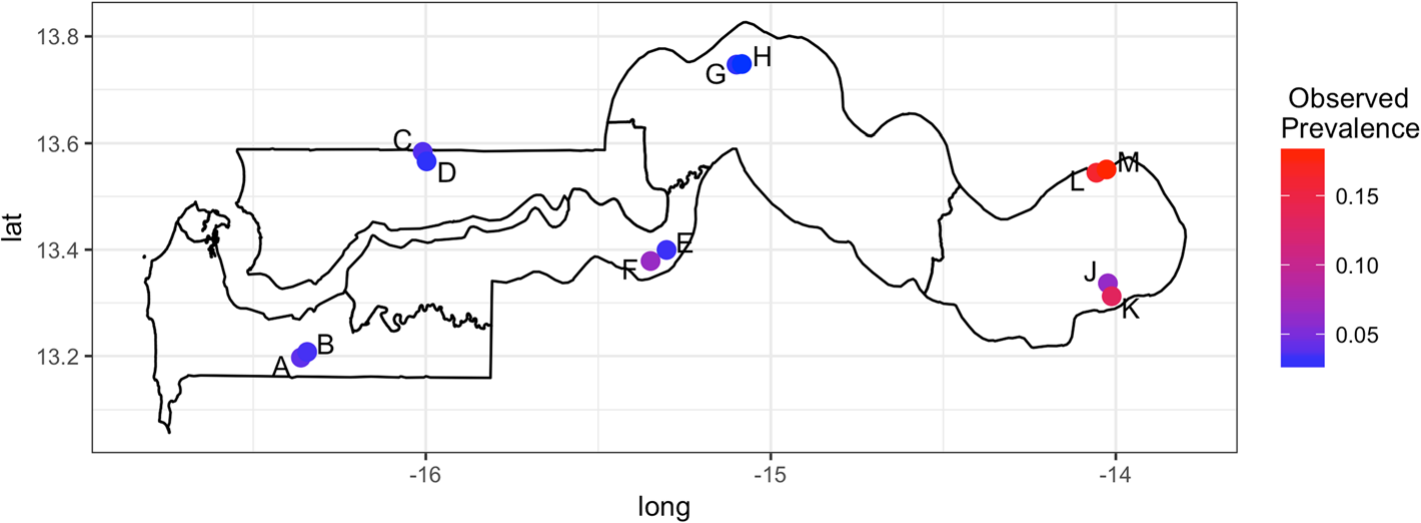
Map of The Gambia showing the location of the 12 study villages. The study villages are represented as *circles* and labeled A–H and J–M. The circles are *colored* according to the overall observed malaria prevalence

Malaria transmission in The Gambia is highly seasonal and occurs during and soon after the rainy season, typically between August and December. Epidemiological data from the study cohort has been recently described [25]. Briefly, monthly blood samples were collected during the 2013 and 2014 malaria transmission seasons (June–December) from all people residing in every household in the study villages (Fig. 1). An additional blood sample was collected during the dry season, in April 2014. Village pairs were approximately 1–3 km apart and were considered as discrete spatial units. Populations ranged between 100 and 700 individuals per village, and all residents were included in the study. All households were geo-located. The number of households per village ranged from 13 to 69, and the distance between households within a village ranged from 0.4 to a maximum of 986.8 m (Table 1). Furthermore, one round of mass drug administration (MDA) with dihydroartemisinin-piperaquine was carried out in June 2014.

**Table 1:** Key characteristics of study villages including demographics and the observed malaria burden

Region	Village	No. people	No. HH	Distance (meters) between HH (min-max)	Median age (IQR)	Median visits per person (IQR)	No. observed infections	Observed PCR prevalence
West Coast	A	670	68	16.6–986.8	13 (6–29)	10 (6–12)	240	0.039
	B	202	23	13.1–360.6	14 (7–32)	10 (6–12)	60	0.033
North Bank	C	273	19	4.7–191.2	12 (4–26)	13 (10–13)	107	0.036
	D	461	30	2.9–327.8	13 (5–27)	11 (5–12)	121	0.029
Lower River	E	112	10	22.7–179.3	13 (8–26)	12 (8–13)	34	0.031
	F	567	69	12.6–776.0	13 (5–27)	9 (4–11)	281	0.064
Central River	G	480	25	2.4–234.8	14 (5–30)	11 (7–12)	135	0.029
	H	204	13	0.4–196.8	13 (5–28)	10 (5–12)	45	0.026
Upper River South	J	418	28	8.1–216.0	14 (6–30)	9 (7–11)	224	0.062
	K	804	42	6.7–550.4	12 (5–27)	8 (6–10)	845	0.134
Upper River North	L	258	13	16.6–253.5	15 (6–26)	11 (9–12)	440	0.164
	M	217	20	8.1–242.8	16 (7–25)	10 (6–12)	345	0.183

*HH* household, *IQR* interquartile range

Finger prick blood samples were collected on filter paper for identification of *Plasmodium falciparum* infections using polymerase chain reaction (PCR). All febrile individuals (auxiliary temperature ≥ 37.5 °C or history of fever in the last 24 h) were screened for malaria by rapid diagnostic test (RDT), and if positive they were treated with artemether-lumefantrine according to national guidelines. A study nurse was stationed within each village and recorded all malaria episodes between monthly visits, including administering an RDT and collecting a blood sample on filter paper.

Malaria parasites are transmitted to humans via the bite of an infected *Anopheles* mosquito and can be directly measured using the entomological inoculation rate (EIR) [26, 27]. The *P. falciparum* parasite rate (*Pf*PR) is a known correlate to EIR; it provides a measure of transmission intensity and is a more operationally feasible metric to generate [28]. Using PCR infection as the dependent variable as a proxy for transmission intensity, geostatistical analysis was conducted using the PrevMap package in R (v3.3.2) to determine the predicted malaria prevalence per household per month within each village accounting for spatial autocorrelation as well as temporal trends [29]. A Bayesian geostatistical probit model was used to predict the spatial variation in malaria parasite prevalence within each village. More details on the model specification are provided in Additional file 1. Because the cohort was a full population sample, no interpolation at unsampled locations was required. Predicted prevalence per household was estimated using the median of the posterior distribution, and maps of the combined and monthly predicted prevalence were generated.

Models were adjusted for sample date, distance to road, distance to river, and mean monthly rainfall. The distance to river and road variables were determined by extracting the relevant features from pan-sharpened Landsat 8 imagery and using the gDistance function in the rgeos package [30] to estimate the straight-line distance in kilometers. Monthly rainfall was obtained from weather stations located in each of the six study regions across the country.

The observed overdispersed distribution of infection counts has been used to support the notion of malaria hotspots [1]. However, it is possible that the skewed distribution is due to measurement bias in how infections are defined. For example, PCR-detected infections were not treated in this study (until becoming symptomatic and detectable by RDT) and could represent an infection from a single infectious bite or repeated inoculations within the same individual until treatment is sought. For example, by considering each time point where a PCR infection is detected as unique would lead to counting a single infection detected at 5 sequential time points as 5 unique infections instead of 1, thereby driving the observed overdispersion. To demonstrate the degree of potential measurement bias as an alternative explanation to the skewed patterns of case counts, two different approaches for counting were employed. The first was to consider each time point when a PCR positive result was recorded as a unique infection irrespective of whether there was a confirmed treatment in between sampling. The second approach considered any infections detected at sequential time points as the same infection unless the individual had been treated for malaria as part of the study. Any negative sample between two PCR positive samples in a non-treated individual was assumed to be a false negative and considered as a single infection. Any subsequent infection detected after a known treatment event (e.g., symptomatic and RDT positive, or participated in the MDA) was considered as a new infection.

## Results

In total, 41,548 monthly observations were available from 360 households across 14 sampling time points. The size of households ranged from a single person to 78 individuals, and the residents had a similar age distribution between villages (Table 1). The aggregated infection prevalence across the study period ranged from 2.6 to 18.3% across the 12 villages (Fig. 1). During the 2-year study period, 2877 samples were positive for malaria infection, with substantial heterogeneity between villages. The lowest transmission village recorded 34 infections in 10 households, whereas the village with the highest transmission had 845 infections in 42 households (Table 1). Across all time points, 12.5% (45/360) households did not record a single infection, while the number of households without any infection varied from 0 in village L to 12 in village F. Household-level observed monthly incidence ranged from 0 to 0.50 infection per person (interquartile range (IQR) = 0.02–0.10) across the sampling months.

**Fig. 2:**
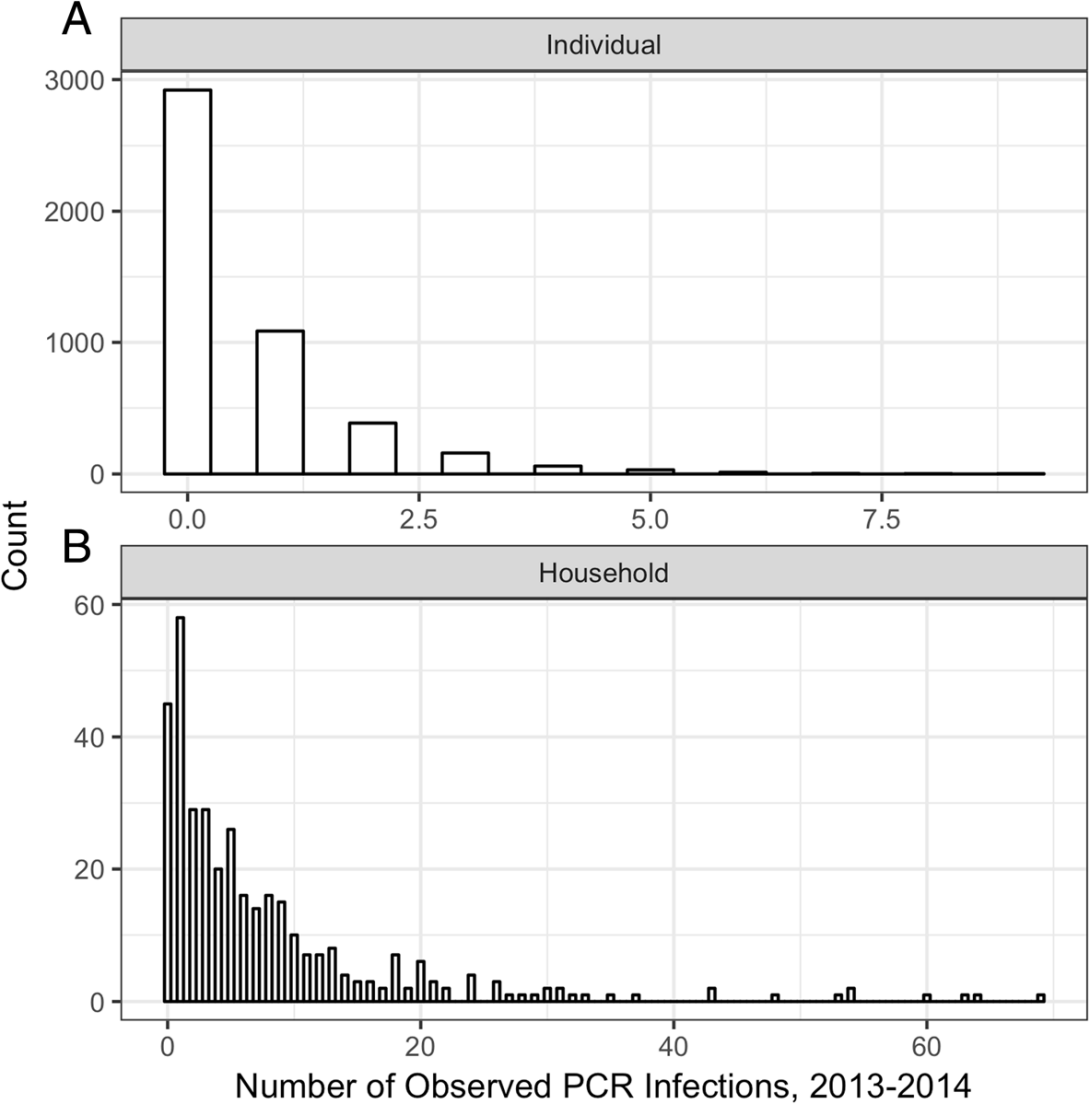
Frequency distributions of malaria infections in the study population. Frequency of number of observed PCR positive infections **a** per individual and **b** per household

**Fig. 3:**
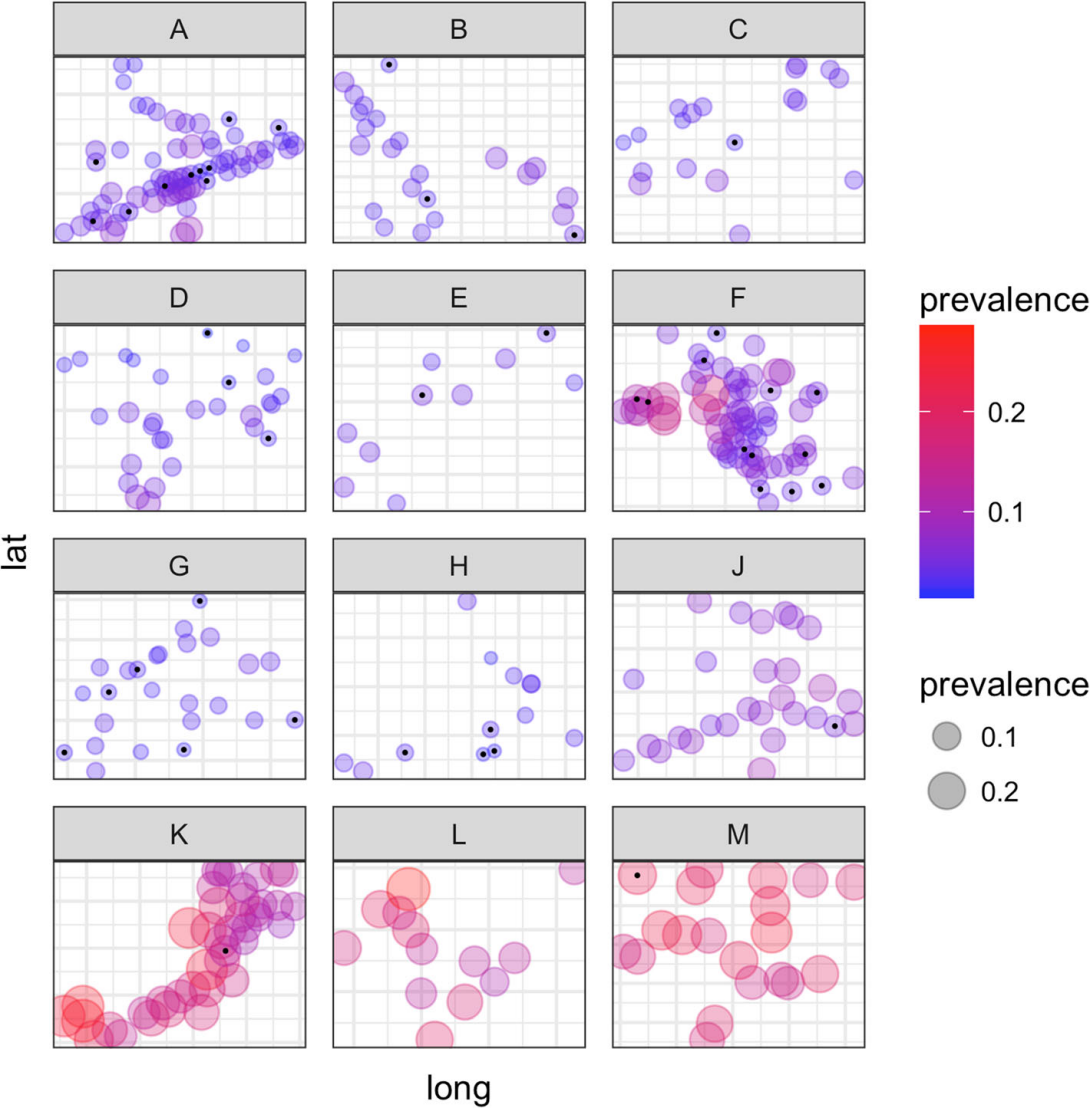
Overall predicted PCR prevalence per household (*circles*), per village (panels **a**-**h**, **j**-**m**, corresponding to the village code) according to the spatio-temporal model. The *size* and *color* of the circles are scaled according to prevalence. The *black dots* identify those households with zero malaria infections recorded during the study


The overall number of observed infections per individual (Fig. 2a) and per household (Fig. 2b) exhibit the expected overdispersion pattern, illustrating the considerable heterogeneity in malaria exposure experienced by this population. Results of the geostatistical model exhibited 100 m as the range of spatial autocorrelation, suggesting that village pairs were discrete transmission units. However, the geostatistical model failed to provide evidence of a pronounced spatial pattern within villages at either low or high transmission intensities (Fig. 3; see Additional file 1 for model output). Across all villages, only a single village (Fig. 3, village F) showed a pattern of high burden households grouping together. When the predicted household-level prevalence is plotted over time, there is no evidence that infection dynamics around high burden households exhibit a regular pattern around neighboring households at the monthly time step; the patterns appear stochastic (Additional file 2). Furthermore, as a group of high burden households was only evident in a single village with moderate transmission levels, the presence of hotspots within villages does not appear to be associated with transmission intensity. The spatial patterns were similar irrespective of whether an infection was symptomatic or asymptomatic (Additional file 3) [25].

Additional file 2:Monthly predicted PCR prevalence per household (*circles*) for all study villages (*panels*) according to the spatio-temporal model. The *size* and *color* of the *circles* are scaled according to prevalence. Each household is identified by a black dot. Households with a predicted PCR prevalence between 0 and 1% are identified in greyscale. (MP4 285 kb)


**Fig. 4:**
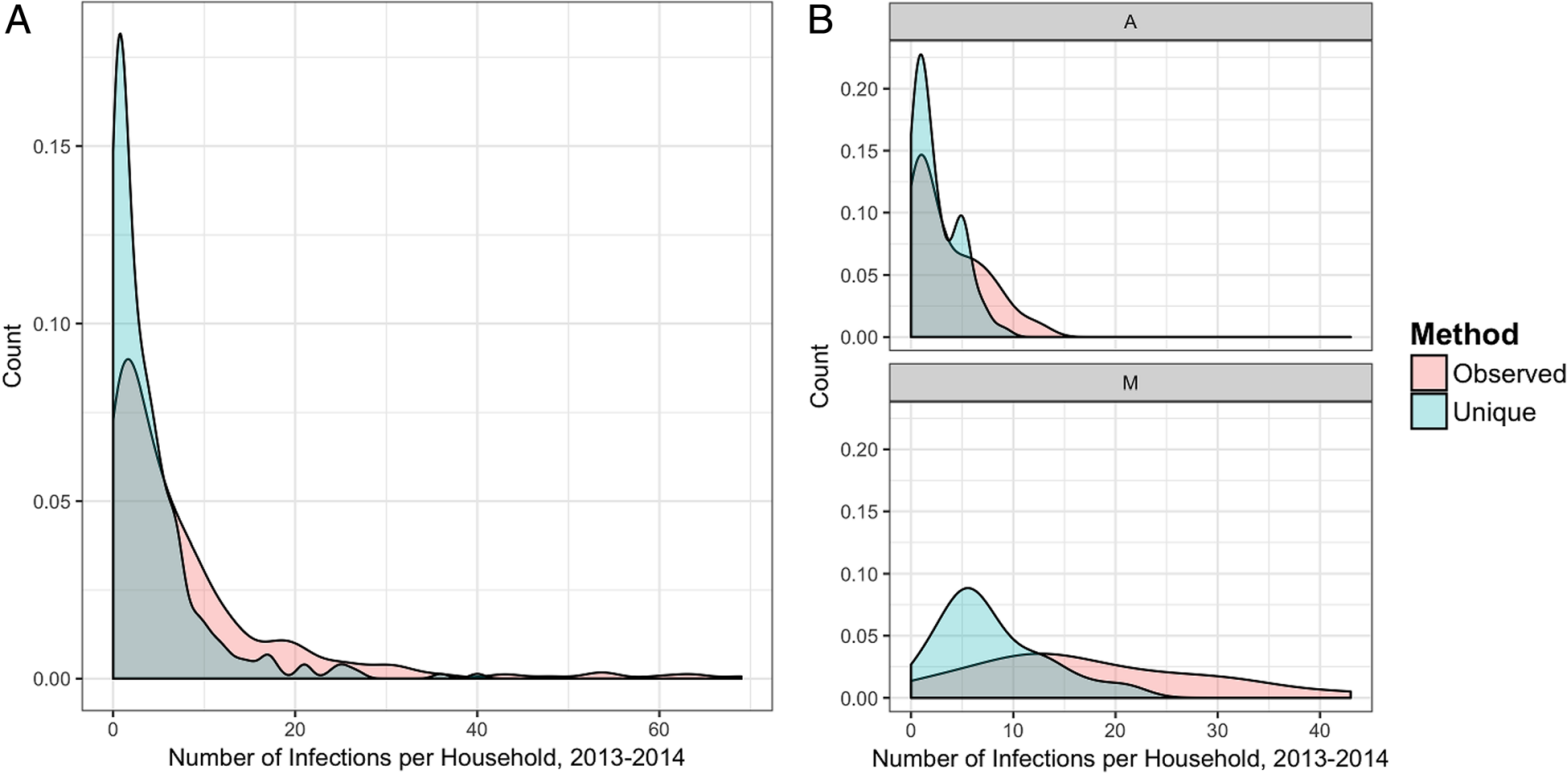
Density plot for the number of infections per compound according to the two definitions tested. Distributions according to definitions are provided for **a** the combined data and **b** an example of a low (A) and high (M) transmission village. The *red curves* show the distribution if each time point with an infection is counted as new. The *blue curves* show the distribution of unique infections assuming an infection is only counted as new if there is evidence of treatment at a prior time point

As a consistent spatio-temporal dynamic of malaria around high burden households was not observed, the next step was to explore alternative explanations for the overdispersion pattern of malaria burden in the study population. The first explanation examined was measurement bias in how infections were defined. If we consider the most conservative definition and assume only new infections as those after a recorded treatment event, the distribution becomes less skewed, with fewer households experiencing multiple malaria episodes (Fig. 4a). As expected, the differences between methods for counting infections are more pronounced in high transmission settings (Fig. 4b, village M) compared to low transmission settings (Fig. 4b, village A). Although neither method of counting infections is expected to fully capture the number of “true” infections experienced in the population, the heterogeneity in malaria burden was still present despite the most extreme definition of counting infections being applied.

**Fig. 5:**
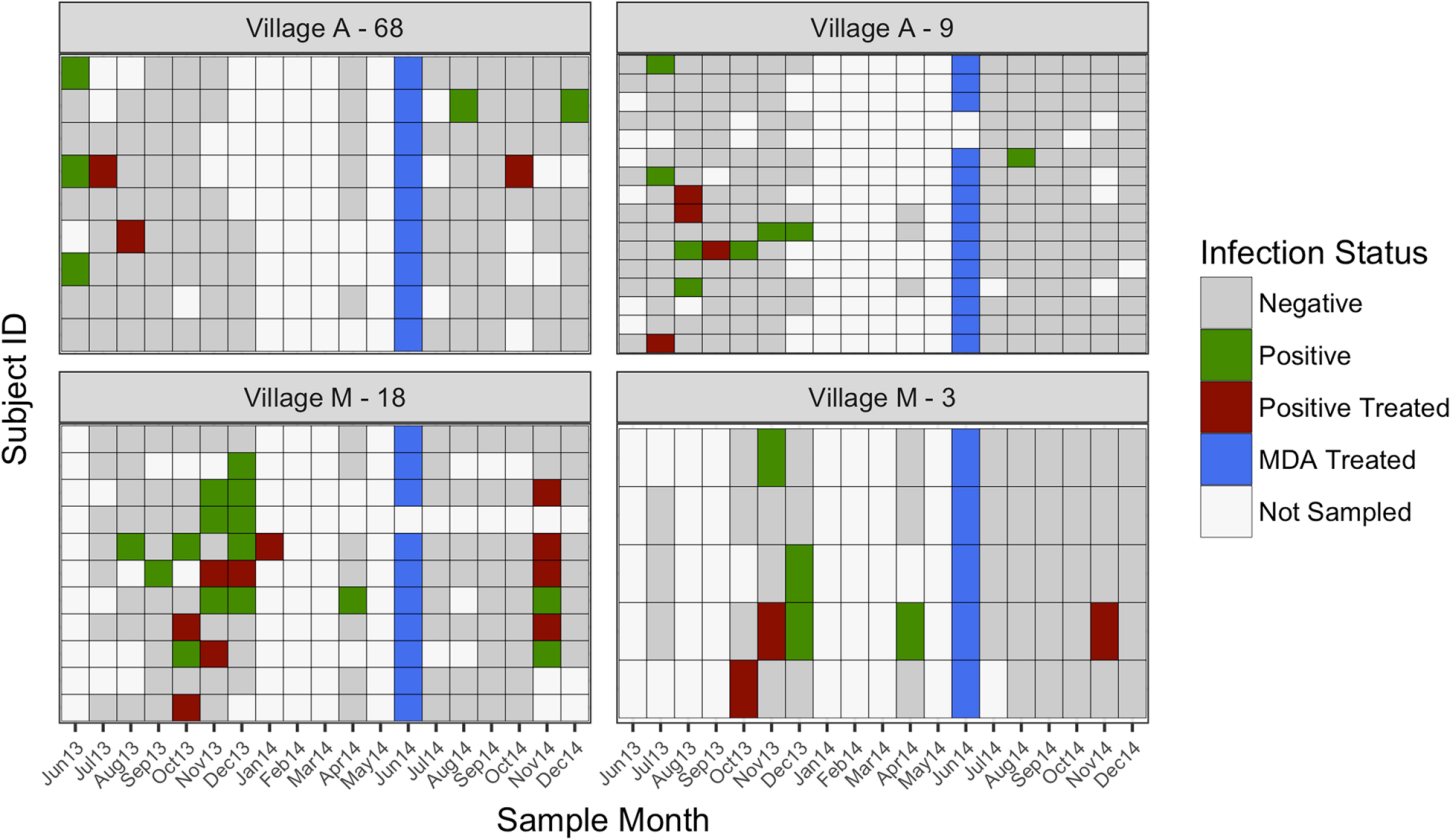
Heat maps showing within-household transmission dynamics. Heat maps showing within-household transmission dynamics in a low transmission village (*village A*) and a high transmission village (*village M*). Each *grid* represents a household with each individual residing within the household shown in the *rows*. Each *column* within each grid represents a sampling month starting in June 2013 through December 2014. The *color* of each grid cell represents their infection and/or treatment status at that time point. Infection status is defined by those who are PCR positive with treatment being administered when there was a symptomatic infection confirmed by RDT in the field or the mass drug administration (MDA) administered between transmission seasons (June 2014)

The second explanation for the observed heterogeneity in malaria that we explored was to consider the household as the relevant spatial unit of transmission. Patterns of infections appearing within households suggested that three scenarios are evident: there are cases when several individuals are infected within the same month, there are cases of infections appearing the month after another individual within the household becomes infected, and there are cases of stochastic introductions (Fig. 5). All patterns were observed in households in both the low (Fig. 5; village A) and high (Fig. 5; village M) transmission settings. However, parasite genetic data is required to confirm this hypothesis. See Additional file 4 for heat maps showing transmission dynamics within all study households.

## Discussion

Heterogeneity in malaria burden is an inherent aspect of transmission, rooted in complex interactions between environmental, vector, and individual characteristics [9, 21, 31]. However, evidence on the importance of the observed heterogeneity within a village in maintaining or fueling transmission, consistent with the concept of hotspots, is required to support the use of such a strategy as part of control or elimination programs. In this study, we explored spatio-temporal trends of malaria transmission intensity to see if it shifted from high burden households to the surrounding area. Though high burden households within villages exist, they were not consistently the same, and the risk of malaria was not observed to spread from high to low burden households at the monthly timescale. Together, these findings suggest that the relevant operational unit for targeting transmission in this setting is the household or the entire village, depending on the program goals and interventions being employed.

As heterogeneity in malaria infections was observed in the data, we next explored non-spatial factors that could be driving the pattern. In this study, participants were only treated if they had a symptomatic, RDT positive infection or participated in the MDA. Therefore, we hypothesized that each observed infection is unlikely to represent a unique infection event, and the overdispersion in burden may be partly driven by measurement bias. Assuming that new infections are only those identified after documented antimalarial treatment decreased but did not eliminate the observed heterogeneity. The “extreme” assumptions we used, namely that all detected infections are new ones or that new infections are only those occurring after treatment, are unlikely to represent the true number of infection events, as individuals may have cleared them spontaneously, received treatment outside of the study, or experienced superinfections [32–34]. Being able to account for superinfections and identify the role of these individuals in fueling onward transmission would help refine methods for counting new or incident infections and determining which infections matter for maintaining transmission intensity [35]. The true incidence likely falls somewhere in between the two estimates used, but measurement bias is unlikely to contribute substantially to the levels of heterogeneity detected.

We next explored the extent to which transmission occurs within the household as a possible explanation for the observed overdispersion. Household-level risk has been identified in other settings whereby individuals residing within an infected house are more likely to also be or become infected [17, 25, 36, 37]. However, it is not known whether the increased burden is due to the aggregation of factors that increase risk of infection or because the household itself is the unit of transmission. In this setting, we observed sequential infections within households where new household members became infected in the month after the initial introduced infection. This pattern suggests that within-household transmission is plausible and supports the use of reactive case detection strategies, where households of any confirmed infection are visited and screened and/or treated for malaria to capture additional cases expected within households of index cases [17, 38]. Based on the limited spreading pattern observed, including neighboring households or those within a specific radius around index households would not be recommended in this setting. Also, a reactive approach for targeting residual infections within households is not likely to be appropriate in all settings. This is particularly true for those settings where transmission occurs outside of the household, for example, in forests, as is common in Southeast Asia [24, 39]. Furthermore, given the stochastic nature of infections across all villages, a reactive approach may not contribute to a reduction of transmission but may contribute to infections averted in household members, particularly if a drug with a longer prophylactic period is used. Given that all villages in this study are capable of supporting transmission and would therefore be considered as “active” according to the World Health Organization (WHO) definition of foci, one could argue that targeting the whole village population with interventions may be more appropriate as a way to accelerate malaria elimination [40].

It is possible that hotspots do exist and fuel transmission within foci, but it was not observed in this setting. It is unlikely that infections were missed, as routine sampling occurred every month during the transmission season with a study nurse capturing episodes between regular visits. Although the monthly time step was selected as it would account for the intrinsic and extrinsic incubation periods, it is possible that this temporal scale was not optimal or the monthly aggregated datasets too small to detect the spreading of infections between households. The treatment of detectable infections as part of both the passive and active screenings may have altered or masked spatio-temporal patterns. However, the expected rate of treatments required to interrupt transmission is much higher than was administered as part of routine surveillance. Secondly, the spatio-temporal patterns observed pre- and post-MDA were similar, despite the magnitude of transmission intensity being lower in the second year. Therefore, the role of treatment likely had a minimal impact on the ability to observe any patterns. Alternative spatio-modeling approaches such as point pattern or dispersion models may have yielded different results. However, the number of points per village limited any point-based analysis, and understanding whether infections cluster would not directly address the question of interest. Incorporating the parasite genetic data into this analysis to track infections within and between households may help us understand the extent of within- and between-household transmission dynamics [41]. The detailed genetic data required for this analysis was not available. However, recent work supports the notion of microepidemiological clustering of parasite strains [33]. Next, the non-response bias experienced in this 2-year cohort may have masked any hotspot dynamics. It is possible that the individuals missed could have better illustrated any spreading between households. However, the participation rate across all villages was reasonably high and was consistent between villages, so although possible, we do not consider this as likely.

## Conclusions

Approaches for more efficient targeting of malaria control and elimination activities have shifted to incorporating spatial dynamics of transmission and identifying lingering foci. Although hotspots fueling malaria transmission within a village or foci are biologically plausible, the limited evidence in field settings puts their role in sustaining transmission into question. The results presented here further support this shift in thinking [40, 42]. This population-level cohort in 12 villages across The Gambia showed that there is considerable heterogeneity in transmission both within and between study villages. Our results suggest that spatio-temporal patterns of malaria risk are stochastic at all endemicities and are inconsistent with the idea of hotspots fueling malaria transmission. Transmission was more likely to occur within households in this setting, supporting the use of reactive case detection strategies targeting the household only or to target the entire village as a focus, but not an approach targeting hotspots with the goal of interrupting transmission from high to low burden areas.

## Additional files

Additional file 1: Description of the geostatistical model including outputs. (PDF 181 kb)

Additional file 3: Results of analysis stratified by whether an infection was symptomatic or asymptomatic. Symptomatic infections were those individuals with fever and a confirmed infection by rapid diagnostic test (RDT), whereas asymptomatic infections were those who were febrile but RDT negative and positive by polymerase chain reaction (PCR) or were afebrile and positive for malaria by PCR. The distribution of prevalence of infection type per month per village as well as the predicted monthly prevalence of each infection type per village are shown. (DOCX 4478 kb)

Additional file 4: Heat maps for all villages, removing households with no infections and individuals with 5 or fewer time points sampled. Each *panel* represents a household, and each *row* within the panel represents an individual residing in that household. Individuals are ordered by increasing age with the youngest on top within each panel. Each *column* within each *grid* represents a sampling month starting in June 2013 through December 2014. (DOCX 4496 kb)

## Abbreviations

IQR: Interquartile range
MDA: Mass drug administration
PCR: Polymerase chain reaction
RDT: Rapid diagnostic test
