# Supplementary material for: Do hotspots fuel malaria transmission: a village-scale spatio-temporal analysis of a 2-year cohort study in The Gambia
Source: BMC Med. 2018 Sep 14;16:160. doi: 10.1186/s12916-018-1141-4 (PMC6137946; doi:10.1186/s12916-018-1141-4)
Supplement: Supplementary file 2 — Description of the geostatistical model including outputs. (PDF 181 kb) [file 12916_2018_1141_MOESM1_ESM.pdf]

Additional File 1: Geostatistical analysis

Model formulation

Let Y_{ij} denote a random binary outcome associated with the j-th individual at the household location x_i and month t_i, taking value 1 for a positive PCR test for *Plasmodium falciparum* and 0 otherwise. Conditionally on a spatial Gaussian process $S(x_i)$, we model the probability of a positive PCR test, $p_j(x_i, t_i)$, using a probit-linear regression, i.e.

$$\Phi\left\{p_j(x_i, t_i)\right\}^{-1} = \alpha + \sum_{k=1}^{p} \beta_k d_k(x_i, t_i) + \gamma e_{ij} + S(x_i), \tag{1}$$

where e_{ij} is the age of the sampled individual, the $d_k(x_i, t_i)$ are a set of spatio-temporally referenced covariates (see Table 1) and (α, β, γ) are regression coefficients to be estimated.

We model $S(x)$ as an isotropic and stationary Gaussian process with covariance function given by

$$\text{cov}\{S(x), S(x')\} = \sigma^2 \exp\{-\|x - x'\|/\phi\}$$

where σ^2 is the variance of $S(x)$ and ϕ is a scale parameter which regulates how fast the spatial correlation decays to 0 for increasing distance.

We use Bayesian methods of inference with following set of independent priors:

- $\alpha \sim N(0, 10^3)$;
- $\beta_k \sim N(0, 10^3)$, $k = 1, \ldots, 4$;
- $\gamma \sim N(0, 10^3)$;
- $\log\{\sigma^2\} \sim (0, 2.5)$;
- $\log\{\phi\} \sim (\log 100, 1)$.

We fit the model using a data-augmentation approach (Holmes & Held, 2011) implemented in the `PrevMap` R package (Giorgi & Diggle, 2017). Table 2 reports the posterior point and interval estimates for the model parameters.

Table 1: List of the spatio-temporally referenced explanatory variables.

Regression coefficient	Covariate
β_1	Rainfall (mm)
β_2	Distance from the closest waterway (m)
β_3	Distance from the main road (m)
β_4	Binary indicator of post-MDA year (1=yes, 0=no)

References

Giorgi, E. & Diggle, P. J. (2017). PrevMap: An R package for prevalence mapping. *Journal of Statistical Software* **78**, 1–29.

Holmes, C. & Held, L. (2011). Response to van der lans. *Bayesian Anal.* **6**, 357–358.

Table 2: Posterior summaries from the model in (1), including the posterior mean and 95% credible intervals (CI).

	Poterior mean	95% CI
α	237.696	(193.844, 285.425)
β_1	-0.002	(-0.003, -0.001)
$\beta_2 \times 10^3$	-8.342	(-10.526, -6.401)
$\beta_3 \times 10^3$	1.030	(0.736, 1.310)
$\beta_4 \times 10^3$	-3.339	(-6.301, -0.729)
γ	-0.119	(-0.143, -0.097)
σ^2	0.070	(0.042, 0.104)
ϕ	102.506	(47.099, 192.768)
