# Supplementary material for: Do hotspots fuel malaria transmission: a village-scale spatio-temporal analysis of a 2-year cohort study in The Gambia
Source: BMC Med. 2018 Sep 14;16:160. doi: 10.1186/s12916-018-1141-4 (PMC6137946; doi:10.1186/s12916-018-1141-4)
Supplement: Supplementary file 3 — Results of analysis stratified by whether an infection was symptomatic or asymptomatic. Symptomatic infections were those individuals with fever and a confirmed infection by rapid diagnostic test (RDT), whereas asymptomatic infections were those who were febrile but RDT negative and positive by polymerase chain reaction (PCR) or were afebrile and positive for malaria by PCR. The distribution of prevalence of infection type per month per village as well as the predicted monthly prevalence of each infection type per village are shown. (DOCX 4478 kb) [file 12916_2018_1141_MOESM3_ESM.docx]

Additional File 3

**Results of Asymptomatic and Symptomatic Only, 2013 Analysis**

**Distribution of raw prevalence data by village per month for 2013**

Asymptomatic

Symptomatic

**Spatio-temporal monthly predicted prevalence per village by infection type**

Village A

Asymptomatic

Symptomatic

Village B

Asymptomatic

Symptomatic

Village C

Asymptomatic

Symptomatic

Village D

Asymptomatic

Symptomatic

Village E

Asymptomatic

Symptomatic

Village F

Asymptomatic

Symptomatic

Village G

Asymptomatic

Symptomatic

Village H

Asymptomatic

Symptomatic

Village J

Asymptomatic

Symptomatic

Village K

Asymptomatic

Symptomatic

Village L

Asymptomatic

Symptomatic

Village M

Asymptomatic

Symptomatic
