# Supplementary material for: Do hotspots fuel malaria transmission: a village-scale spatio-temporal analysis of a 2-year cohort study in The Gambia
Source: BMC Med. 2018 Sep 14;16:160. doi: 10.1186/s12916-018-1141-4 (PMC6137946; doi:10.1186/s12916-018-1141-4)
Supplement: Supplementary file 4 — Heat maps for all villages, removing households with no infections and individuals with 5 or fewer time points sampled. Each panel represents a household, and each row within the panel represents an individual residing in that household. Individuals are ordered by increasing age with the youngest on top within each panel. Each column within each grid represents a sampling month starting in June 2013 through December 2014. (DOCX 4496 kb) [file 12916_2018_1141_MOESM4_ESM.docx]

Additional file 4

Individual Level Infection Patterns within Household Heat Maps for All Villages

**Village A**

**Village B**

**Village C**

**Village D**

**Village E**

**Village F**

**Village G**

**Village H**

**Village J**

**Village K**

**Village L**

**Village M**
